# Hypothyroidism *in utero* stimulates pancreatic beta cell proliferation and hyperinsulinaemia in the ovine fetus during late gestation

**DOI:** 10.1113/JP273555

**Published:** 2017-03-13

**Authors:** Shelley E. Harris, Miles J. De Blasio, Melissa A. Davis, Amy C. Kelly, Hailey M. Davenport, F. B. Peter Wooding, Dominique Blache, David Meredith, Miranda Anderson, Abigail L. Fowden, Sean W. Limesand, Alison J. Forhead

**Affiliations:** ^1^ Department of Biological and Medical SciencesOxford Brookes UniversityOxfordOX3 0BPUK; ^2^ Department of Physiology, Development and NeuroscienceUniversity of CambridgeCambridgeCB2 3EGUK; ^3^ School of Animal and Comparative Biomedical SciencesUniversity of ArizonaTucsonAZ85721USA; ^4^ School of Animal BiologyUniversity of Western Australia6009CrawleyAustralia

**Keywords:** fetal programming, insulin, islet cell, pancreas, thyroid hormone

## Abstract

**Key points:**

- Thyroid hormones are important regulators of growth and maturation before birth, although the extent to which their actions are mediated by insulin and the development of pancreatic beta cell mass is unknown.
- Hypothyroidism in fetal sheep induced by removal of the thyroid gland caused asymmetric organ growth, increased pancreatic beta cell mass and proliferation, and was associated with increased circulating concentrations of insulin and leptin.
- In isolated fetal sheep islets studied *in vitro*, thyroid hormones inhibited beta cell proliferation in a dose‐dependent manner, while high concentrations of insulin and leptin stimulated proliferation.
- The developing pancreatic beta cell is therefore sensitive to thyroid hormone, insulin and leptin before birth, with possible consequences for pancreatic function in fetal and later life.
- The findings of this study highlight the importance of thyroid hormones during pregnancy for normal development of the fetal pancreas.

**Abstract:**

Development of pancreatic beta cell mass before birth is essential for normal growth of the fetus and for long‐term control of carbohydrate metabolism in postnatal life. Thyroid hormones are also important regulators of fetal growth, and the present study tested the hypotheses that thyroid hormones promote beta cell proliferation in the fetal ovine pancreatic islets, and that growth retardation in hypothyroid fetal sheep is associated with reductions in pancreatic beta cell mass and circulating insulin concentration *in utero*. Organ growth and pancreatic islet cell proliferation and mass were examined in sheep fetuses following removal of the thyroid gland *in utero*. The effects of triiodothyronine (T_3_), insulin and leptin on beta cell proliferation rates were determined in isolated fetal ovine pancreatic islets *in vitro*. Hypothyroidism in the sheep fetus resulted in an asymmetric pattern of organ growth, pancreatic beta cell hyperplasia, and elevated plasma insulin and leptin concentrations. In pancreatic islets isolated from intact fetal sheep, beta cell proliferation *in vitro* was reduced by T_3_ in a dose‐dependent manner and increased by insulin at high concentrations only. Leptin induced a bimodal response whereby beta cell proliferation was suppressed at the lowest, and increased at the highest, concentrations. Therefore, proliferation of beta cells isolated from the ovine fetal pancreas is sensitive to physiological concentrations of T_3_, insulin and leptin. Alterations in these hormones may be responsible for the increased beta cell proliferation and mass observed in the hypothyroid sheep fetus and may have consequences for pancreatic function in later life.

## Key points

- Thyroid hormones are important regulators of growth and maturation before birth, although the extent to which their actions are mediated by insulin and the development of pancreatic beta cell mass is unknown.
- Hypothyroidism in fetal sheep induced by removal of the thyroid gland caused asymmetric organ growth, increased pancreatic beta cell mass and proliferation, and was associated with increased circulating concentrations of insulin and leptin.
- In isolated fetal sheep islets studied *in vitro*, thyroid hormones inhibited beta cell proliferation in a dose‐dependent manner, while high concentrations of insulin and leptin stimulated proliferation.
- The developing pancreatic beta cell is therefore sensitive to thyroid hormone, insulin and leptin before birth, with possible consequences for pancreatic function in fetal and later life.
- The findings of this study highlight the importance of thyroid hormones during pregnancy for normal development of the fetal pancreas.

**Abbreviations**
dGA: days of gestation
EdU: 5‐ethynyl‐2′‐deoxyuride
FBS: fetal bovine serum
IGF: insulin‐like growth factor
KRB: Krebs‐Ringer Buffer
MAFA: musculoaponeurotic fibrosarcoma oncogene family A
pHis: phospho‐histone H3
T_3_: triiodothyronine
T_4_: thyroxine
TR: thyroid hormone receptors
TX: thyroidectomy

## Introduction

Development of the endocrine pancreas and the onset of insulin secretion before birth are essential for normal growth of the fetus. Insulin is a potent growth factor *in utero*: intrauterine growth‐retardation is associated with low circulating insulin concentration in the human fetus (Harding & Johnston, 1995) and experimental insulin deficiency causes growth‐retardation in the ovine fetus (Fowden *et al*. 1989). Late gestation is a critical period for beta cell proliferation in the fetal pancreas and the establishment of beta cell mass at birth. Importantly, development of the endocrine pancreas is sensitive to changes in nutrient availability and circulating hormone levels *in utero* with long‐term consequences for carbohydrate metabolism and the risk of type 2 diabetes in adulthood (Fowden & Hill, 2001; Portha *et al*. 2011; Gatford & Simmons, 2013).

Thyroid hormones, thyroxine (T_4_) and triiodothyronine (T_3_), also play an important role in the control of growth, metabolism and development of the fetus, especially over the prepartum period when T_3_ concentrations rise in the fetal circulation (Fisher & Polk, 1989; Forhead & Fowden, 2014). Congenital hypothyroidism affects approximately 1 in 3000 human births every year and causes severe mental and physical retardation if untreated (LaFranchi, 2011). In animal models, experimental hypothyroidism *in utero* impairs fetal growth and maturation of organ systems, including the cardiovascular, nervous and skeletomuscular systems (Finkelstein *et al*. 1991; Lanham *et al*. 2011; Patel *et al*. 2011; Chattergoon *et al*. 2012; Forhead & Fowden, 2014).

The actions of thyroid hormones on growth and development of fetal tissues may be both direct and indirect as they can influence other endocrine systems before birth. Thyroid hormone deficiency in fetal sheep alters the availability of hormones and growth factors in the circulation and tissues, such as leptin and insulin‐like growth factors (IGFs; Forhead *et al*. 2002; O'Connor *et al*. 2007). Interactions between insulin and thyroid hormones in the control of fetal growth and development, however, are poorly understood. Thyroid hormone receptors, TRα1 and TRβ1, are expressed in the embryonic murine pancreas (Aiello *et al*. 2014), and T_3_ promotes beta cell proliferation in human and rodent cell lines (Verga Falzacappa *et al*. 2006; Furuya *et al*. 2010; Kim *et al*. 2014) and in the embryonic murine pancreas in explant culture (Aiello *et al*. 2014). Little is known, however, about the role of thyroid hormones in the development of the endocrine pancreas and secretion of insulin in the fetus during late gestation, a critical period when beta cell proliferation is sensitive to changes in the intrauterine environment (Bouwens & Rooman, 2005; Portha *et al*. 2011).

The aims of the current study were to examine (a) the effects of hypothyroidism *in utero* on pancreatic alpha and beta cell mass in the ovine fetus and (b) the endocrine control of beta cell proliferation *in vitro* using isolated fetal ovine pancreatic islets. It was hypothesised that thyroid hormones promote fetal pancreatic beta cell proliferation *in vitro*, and that thyroid hormone deficiency in the ovine fetus during late gestation causes a decrease in circulating insulin concentration secondary to a reduction in pancreatic beta cell mass. These changes in pancreatic development and insulin secretion may, in turn, be responsible for the impaired body growth seen in the hypothyroid ovine fetus.

## Methods

### Ethical approval

All surgical and experimental procedures were carried out in accordance with UK Home Office legislation and the Animals (Scientific Procedures) Act 1986, after ethical approval by the Animal Welfare and Ethical Review Body of the University of Cambridge, UK.

### Animals

Nineteen pregnant Welsh Mountain ewes of known gestational age and carrying twin fetuses (15 female, 23 male) were maintained on 200 g day^−1^ concentrates with hay and water *ad libitum*. Food, but not water, was withheld from the ewes for 18–24 h before surgery.

### Experimental procedures

At 105–110 days of gestation (dGA; term ∼145 ± 2 days) and under isoflurane anaesthesia (1.5% isoflurane in O_2_/N_2_O), the twin fetuses of each ewe underwent either thyroidectomy (TX) or a sham operation in which the thyroid gland was exposed but not removed (Hopkins & Thorburn, 1972). At surgery, antibiotics were administered to the amniotic cavity of each fetus (600 mg benzylpenicillin in 2 ml of 0.9% saline: Crystapen, Schering‐Plough, Welwyn Garden City, UK). The ewes were given antibiotics (30 mg kg^−1^ i.m. procaine benzylpenicillin; Depocillin, Intervet UK Ltd, Milton Keynes, UK) and an analgesic agent (1 mg kg^−1^ s.c. carprofen; Rimadyl, Vet UK, Thirsk, UK) immediately before the start of surgery, and antibiotic administration was continued daily for 3 days thereafter. The animals were monitored over the recovery period and normally resumed feeding within 24 h of surgery.

At either 129 (*n = *18) or 143 dGA (*n = *20), the fetuses were delivered by Caesarean section under general anaesthesia (20 mg kg^−1^ maternal body weight sodium pentobarbitone i.v.). Umbilical arterial blood samples were collected into EDTA‐containing tubes. Each fetus was weighed and a variety of fetal organs, including the pancreas, were collected after the animal was killed (200 mg kg^−1^ sodium pentobarbitone i.v.).

### Hormone measurements

Umbilical plasma T_3_ and T_4_ concentrations were determined by radioimmunoassay (MP Biomedicals, Loughborough, UK); intra‐assay CVs were 3 and 5%, and minimum levels of detection were 0.14 and 7.0 ng ml^−1^, respectively. Plasma insulin and cortisol concentrations were measured using ELISA kits (insulin: Mercodia, Uppsala, Sweden; cortisol: IBL International, Hamburg, Germany); intra‐assay CVs were both 9%, and minimum levels of detection were 0.05 and 2.5 ng ml^−1^, respectively. Plasma concentrations of leptin, IGF‐I and IGF‐II were determined by radioimmunoassay as previously described (Blache *et al*. 2000; Forhead *et al*. 2011). Intra‐assay CVs were 4–5%, and minimum levels of detection were 0.09, 0.08 and 4.0 ng ml^−1^, respectively. Plasma glucagon levels were not determined as the blood samples were not collected in the appropriate preservative.

### Pancreas stereology and immunohistochemistry

The fetal pancreas was fixed in 4% paraformaldehyde (with 0.2% glutaraldehyde in 0.1 m phosphate buffer) and embedded in paraffin wax. Each pancreas was exhaustively cut into 5 μm sections and, with a random start between 1 and 100, every 100th section was sampled so that at least 10 sections were collected. Pancreatic alpha and beta cells were immunolabelled using mouse monoclonal antibodies against glucagon (1:1000; Sigma, Poole, UK) and insulin (1:1000; Sigma), respectively. Detection was achieved using the Vectastain Elite ABC kit (Vector Laboratories, Peterborough, UK), diaminobenzidene and methyl‐green as a counter‐stain. Digital images were created using a NanoZoomer digital slide scanner (Hamamatsu Photonics, Welwyn Garden City, UK) and analysed blind to the treatment group. Total pancreatic and islet (alpha and beta cell) volumes were determined by Cavalieri's principle using NewCAST stereological software [Visiopharm, Hoersholm, Denmark (Gundersen & Jensen, 1987; Howard & Reed, 2010)]. In each section, 10% of the total tissue was sampled, which was sufficient to provide at least 200 measurements of glucagon or insulin‐positive tissue. Absolute and relative alpha and beta cell masses were calculated using fetal body and pancreas weights. A subset of 20 fetuses (*n = *5 in each treatment group) were analysed for alpha cell mass.

### Pancreatic alpha and beta cell proliferation

Pancreatic alpha and beta cell proliferation rates were determined by dual immunolabelling with guinea‐pig anti‐porcine insulin (1:500; Dako, Carpinteria, CA, USA), mouse anti‐glucagon (1:500; Sigma) and rabbit anti‐phospho‐histone H3 antibodies (pHis; 7.5 μl ml^−1^; Millipore, Temecula, CA, USA), using immunofluorescence procedures as described previously (Limesand *et al*. 2005; Leos *et al*. 2010). Immunocomplexes were detected using anti‐rabbit IgG conjugated to Alexa Fluor‐488 and anti‐guinea‐pig or anti‐mouse IgG conjugated to Alexa Fluor‐594 (1:500; Jackson ImmunoResearch Laboratories, West Grove, PA, USA). Nuclei were stained with DAPI (Vector Laboratories). Both insulin and glucagon antibodies had been validated previously for use in pancreatic islets from fetal sheep (Limesand *et al*. 2013). No staining for insulin or glucagon was observed in pancreatic tissue outside of the islets or in negative control samples incubated without the primary antibody. Images were digitally captured from a Leica DM5500 microscope and analysed using ImageJ. The proportion of mitotic beta cells was determined from a minimum count of 200 phospho‐histone H3 (pHis)/insulin‐positive cells in a minimum count of 3000 insulin‐positive cells on at least four pancreatic sections from each fetus. The number of pHis‐positive alpha cells was too low to reach the minimum required for stereological analysis (<1% total pancreatic tissue).

### Isolation of fetal ovine pancreatic islets

The experiments on isolated fetal ovine islets were performed at the School of Animal and Comparative Biomedical Sciences, University of Arizona, USA. Five Columbia‐Rambouillet ewes carrying twin fetuses (4 female, 6 male) were maintained in compliance with the Institutional Animal Care and Use Committee, University of Arizona, which approved the study. Ewes were fed alfalfa pellets and provided water *ad libitum*. At 137 ± 5 dGA, the animals were killed with i.v. sodium pentobarbitone (86 mg kg^−1^) and phenytoin sodium (11 mg kg^−1^). After delivery of the fetus by Caesarean section, the pancreas was perfused, via the common bile duct, with cold collagenase solution containing Krebs‐Ringer Buffer (KRB), 0.425 mg ml^−1^ collagenase V (Sigma) and 0.2% DNase1 (Roche, Indianapolis, IN, USA). Pancreatic islets were isolated as described previously (Limesand *et al*. 2006; Rozance *et al*. 2006) and transferred into RPMI‐1640 media (Sigma) containing 10% fetal bovine serum (FBS; EquaFetal, Atlas Biologicals, Fort Collins, CO, USA), penicillin‐streptomycin (50 and 100 μg; Sigma) and 2.8 mm glucose. In fetal ovine islets studied *in vitro*, this concentration of glucose is the inflection point for glucose utilisation and oxidation (Limesand *et al*. 2006; Rozance *et al*. 2006). The islets were cultured in 10% FBS to maintain a basal rate of cell division; this was important in order to demonstrate both increased and decreased rates of proliferation in response to hormone treatment.

### Hormone treatment and proliferation assay

Islets from three fetuses were used for each hormone study. At least 50 islets in each Petri dish were incubated with either T_3_ (Sigma), insulin (Humulin‐R, Lilly, Indianapolis, IN, USA) or leptin (Abcam, Cambridge, UK) at three doses: 0.1, 1 and 10 ng ml^−1^. The effects of T_4_ were not assessed as T_3_ is the active form of thyroid hormone. An additional dish of islets incubated with 10 ng ml^−1^ IGF‐I (Sigma) was used as a positive control as IGF‐I is known to stimulate beta cell proliferation (Hogg *et al*. 1993). The islets were subjected to 95% O_2_/5% CO_2_ gas for 5 min in a humidity chamber before incubation at 37°C. After 24 h, the islets were transferred into fresh medium supplemented with hormone and 5‐ethynyl‐2′‐deoxyuride (EdU) at a final concentration of 10 μm. After a total incubation time of 48 h, the islets were fixed in 4% paraformaldehyde and frozen in optimum cutting temperature compound (Tissue Tek, Torrance, CA, USA). Islets were sectioned at −20°C to a thickness of 10 μm at 100 μm intervals and labelled for EdU‐positive cells using the Click‐iT EdU Alexa Fluor‐488 kit (Invitrogen, Grand Island, NY, USA).

### Immunofluorescent staining

Insulin‐positive beta cells were identified by immunostaining with a guinea‐pig anti‐porcine insulin antibody (Dako). Immunocomplexes were detected by polyclonal donkey anti‐guinea‐pig Alexa Fluor‐594 and DAPI was used as a counter‐stain. At least 2500–3000 insulin‐positive cells were identified in at least six sections per treatment group. The numbers of cells positive for both EdU and insulin were expressed as a proportion of the total number of insulin‐positive cells.

### Statistical methods

Data from the studies *in vivo* were analysed by three‐way ANOVA with treatment, gestational age and sex of the fetus as factors (SigmaStat 3.5, Systat Software, San Jose, CA, USA). The sex of the fetus had no significant effect on any of the variables measured; therefore, data from male and female fetuses were combined and analysed by two‐way ANOVA followed by the Tukey *post hoc* test. Data from the studies *in vitro* were assessed by one‐way ANOVA followed by the Dunnett *post hoc* test. Relationships between variables were assessed by linear regression. Significance was accepted at *P *˂ 0.05.

## Results

### Hypothyroidism *in utero* increases circulating insulin and leptin concentrations

In TX fetuses, plasma T_4_ and T_3_ concentrations decreased significantly to below, or close to, the limit of assay detection at both 129 and 143 dGA (*P* < 0.05; Table 1). A developmental rise in plasma T_3_ concentration was observed between 129 and 143 dGA in the sham, but not TX, fetuses (*P* < 0.05; Table 1). Compared to sham fetuses, the TX fetuses had significantly higher plasma concentrations of insulin and leptin at both gestational ages (*P* < 0.05; Table 1). There was no effect of TX on plasma cortisol, IGF‐I or IGF‐II concentrations, but cortisol levels were significantly higher in both groups of fetuses studied at 143 dGA compared to those at 129 dGA (*P* < 0.05; Table 1).

**Table 1:** Mean (±SEM) plasma hormone concentrations and body measurements in sham and TX sheep fetuses at 129 and 143 dGA

	129 dGA		143 dGA	
	Sham (*n = *9)	TX (*n = *9)	Sham (*n = *10)	TX (*n = *10)
**Plasma hormone concentrations**				
T_3_ (ng ml^−1^)	0.28 ± 0.07	ND*	0.65 ± 0.13†	ND*
T_4_ (ng ml^−1^)	81.1 ± 15.6	ND*	83.0 ± 16.7	ND*
Insulin (ng l^−1^)	30.1 ± 9.4	129.4 ± 31.3*	55.7 ± 12.0	167.9 ± 19.3*
Cortisol (ng ml^−1^)	12.6 ± 3.1	9.8 ± 1.5	54.9 ± 12.7†	28.7 ± 3.9†
Leptin (pg ml^−1^)	520 ± 40	980 ± 40*	670 ± 50	1000 ± 10*
IGF‐I (ng ml^−1^)	18.4 ± 1.9	21.2 ± 2.8	27.0 ± 5.7	25.3 ± 3.1
IGF‐II (ng ml^−1^)	102.8 ± 10.4	118.8 ± 5.5	105.3 ± 6.7	106.3 ± 3.8
**Body measurements**				
Body weight (kg)	2.61 ± 0.20	2.49 ± 0.20	3.58 ± 0.23†	3.13 ± 0.13†
Crown–rump length (cm)	43.6 ± 1.1	42.1 ± 1.0	48.3 ± 1.1†	45.7 ± 0.7†
Abdominal circumference (cm)	30.8 ± 0.9	29.6 ± 1.2	34.9 ± 0.9†	33.5 ± 0.7†
**Organ mass (relative to body mass, %)**				
Heart	0.63 ± 0.03	0.52 ± 0.01*	0.61 ± 0.02	0.52 ± 0.02*
Lungs	2.86 ± 0.18	2.36 ± 0.08*	2.55 ± 0.12	2.29 ± 0.15*
Empty stomach	0.81 ± 0.04	0.72 ± 0.02*	0.82 ± 0.02	0.69 ± 0.03*
Small intestines	2.00 ± 0.19	1.35 ± 0.07*	2.34 ± 0.07†	1.36 ± 0.07*
Kidneys	0.60 ± 0.03	0.68 ± 0.04	0.52 ± 0.03	0.63 ± 0.03*
Perirenal adipose	0.39 ± 0.03	0.52 ± 0.1*	0.31 ± 0.03	0.48 ± 0.04*
Pancreas	0.09 ± 0.01	0.09 ± 0.01	0.08 ± 0.01	0.09 ± 0.01
Liver	2.85 ± 0.36	2.73 ± 0.17	2.20 ± 0.07	2.94 ± 0.22

†Significantly different from fetuses in the same treatment group at 129 dGA; ^*^significantly different from sham fetuses at the same gestational age; two‐way ANOVA, *P* < 0.05. ND, not detectable (minimum levels of detection: T_3_ 0.14 ng ml^−1^, T_4_ 7.0 ng ml^−1^).

### Hypothyroidism *in utero* induces asymmetrical growth patterns in fetal organs

There were no differences in body weight, crown–rump length or abdominal circumference between sham and TX fetuses at either gestational age (Table 1). Relative weights of the heart, lungs, stomach and small intestines were significantly lower in TX fetuses compared to sham fetuses at both 129 and 143 dGA (*P* < 0.05; Table 1). In TX fetuses, significant increments were observed in the relative weights of the kidneys (at 143 dGA) and perirenal adipose tissue (at both 129 and 143 dGA; *P* < 0.05; Table 1). There was no effect of TX on the relative mass of the pancreas or liver at either gestational age (Table 1).

### Hypothyroidism *in utero* induces pancreatic beta cell hyperplasia

**Figure 1:**
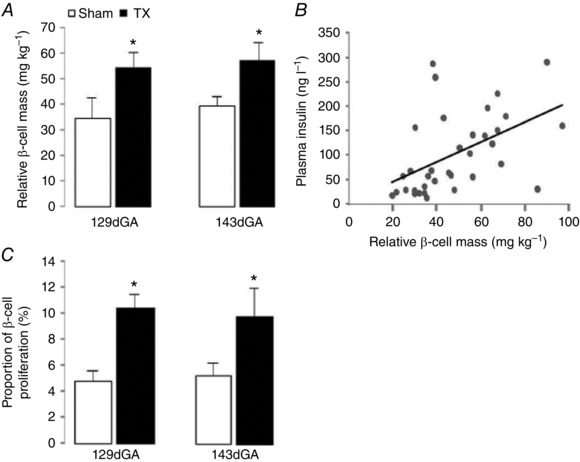
Pancreatic beta cell mass and proliferation in sham and TX fetuses *A* and *C*, mean (±SEM) pancreatic beta cell (*A*) relative mass and (*C*) proliferation rate in sham (white bars) and TX (black bars) sheep fetuses at 129 and 143 dGA. ^*^Significantly different from sham fetuses at the same gestational age, two‐way ANOVA, *P* < 0.05; *n = *8–10 fetuses in each group. *B*, relationship between relative beta cell mass and plasma insulin concentration in sham and TX sheep fetuses at 129 and 143 dGA; *n = *36, *r = *0.50, *P *˂ 0.005.

The volume percentage of beta cells in total pancreatic tissue was significantly greater in TX fetuses compared to sham fetuses at 129 dGA (sham 3.6 ± 0.4% *vs*. TX 6.2 ± 0.5%, *P* < 0.05), but not at 143 dGA (sham 5.1 ± 0.7% *vs*. TX 6.4 ± 0.5%). Absolute beta cell mass tended to be greater in TX fetuses than in sham controls at 129 dGA (sham 92.3 ± 24.1 mg *vs*. TX 144.6 ± 23.5 mg) and 143 dGA (sham 143.9 ± 20.9 mg *vs*. TX 183.4 ± 26.8 mg, *P = *0.08). In sham, but not TX, fetuses at 143 dGA, there was a significant increase in absolute beta cell mass compared to the younger fetuses at 129 dGA (*P* < 0.05). When adjusted for fetal body weight, relative beta cell mass was 30–40% greater in TX compared to sham fetuses at both gestational ages (*P* < 0.05, Fig. 1 *A*). When observations from all fetuses were considered, regardless of treatment and gestational age, plasma insulin concentration was positively correlated with relative pancreatic beta cell mass (*r = *0.50, *n = *36, *P* < 0.005, Fig. 1 *B*). The proportion of proliferating beta cells was 45–55% greater in TX compared to sham fetuses at both 129 and 143 dGA (*P* < 0.05, Fig. 1 *C*). There was no effect of gestational age on beta cell proliferation rate in either treatment group (Fig. 1 *C*).

The proportion of alpha cells in total pancreatic tissue was unchanged in TX compared to control fetuses at 129 dGA (sham 1.4 ± 0.2% *vs*. TX 1.9 ± 0.3%) and 143 dGA (sham 1.6 ± 0.2% *vs*. TX 1.5 ± 0.1%). When adjusted for pancreas weight, there was no effect of thyroidectomy or gestational age on absolute alpha cell mass at either 129 dGA (sham 37.3 ± 7.0 mg *vs*. TX 43.3 ± 6.4 mg) or 143 dGA (sham 41.4 ± 6.9 mg *vs*. TX 44.2 ± 4.1 mg). When adjusted for fetal body weight, there was no change in relative alpha cell mass at either gestational age (129 dGA: sham 12.6 ± 1.9 mg kg^−1^ *vs*. TX 15.1 ± 2.3 mg kg^−1^; 143 dGA sham 11.7 ± 0.9 mg kg^−1^ *vs*. TX 14.5 ± 1.4 mg kg^−1^).

### Thyroid hormone, insulin and leptin alter fetal beta cell proliferation *in vitro*

**Figure 2:**
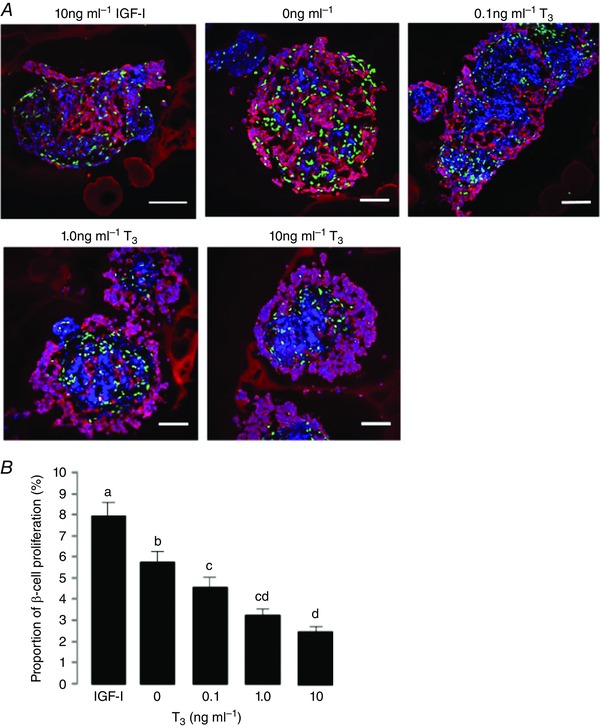
Beta cell proliferation in T_3_ treated islets *A*, immunofluorescent staining to show insulin (red), EdU (green) and DAPI (blue) in fetal ovine pancreatic islets cultured in 10 ng ml^−1^ IGF‐I (positive control) or 0, 0.1, 1.0 and 10 ng ml^−1^ T_3_. Scale bar = 25 μm. *B*, mean (±SEM) proliferation rates in beta cells after incubation with increasing doses of T_3_ compared to 10 ng ml^−1^ IGF‐I. Values with different letters are significantly different from each other, one‐way ANOVA, *P* < 0.05.

**Figure 3:**
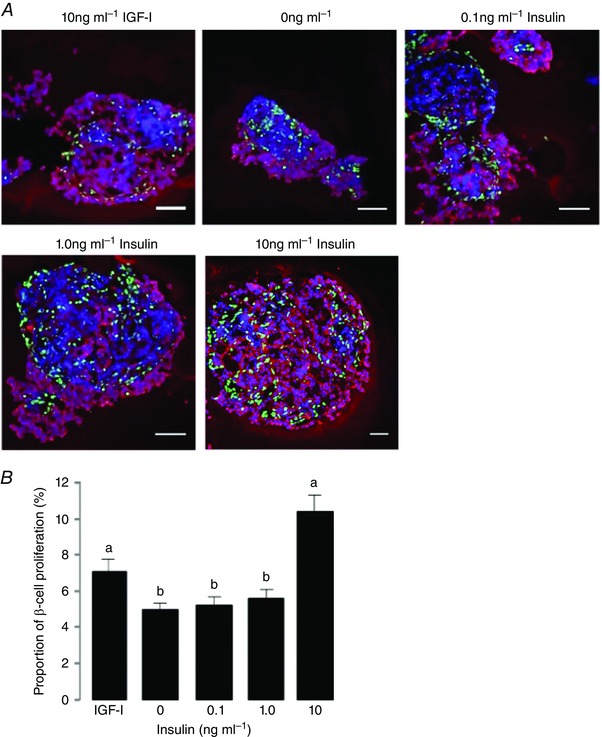
Beta cell proliferation in insulin treated islets *A*, immunofluorescent staining to show insulin (red), EdU (green) and DAPI (blue) in fetal ovine pancreatic islets cultured in 10 ng ml^−1^ IGF‐I (positive control) or 0, 0.1, 1.0 and 10 ng ml^−1^ insulin. Scale bar = 25 μm. *B*, mean (±SEM) proliferation rates in beta cells after incubation with increasing doses of insulin compared to 10 ng ml^−1^ IGF‐I. Values with different letters are significantly different from each other, one‐way ANOVA, *P* < 0.05.

**Figure 4:**
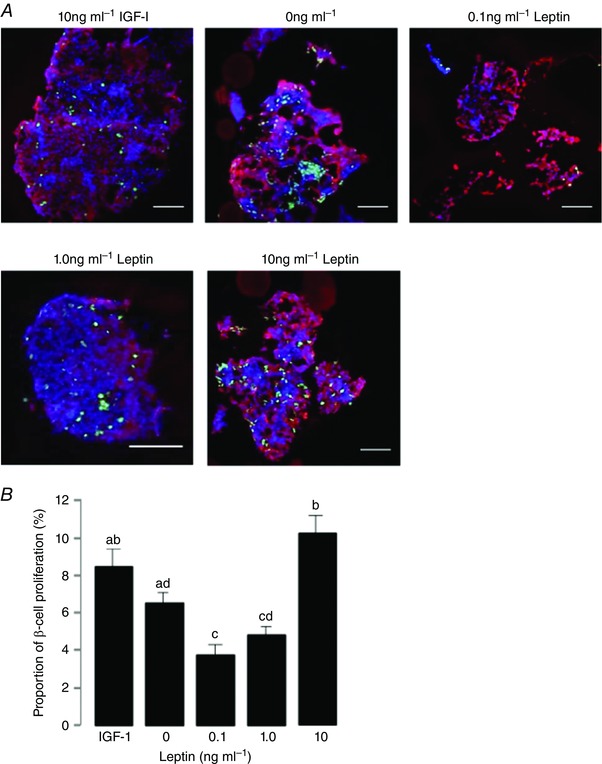
Beta cell proliferation in leptin treated islets *A*, immunofluorescent staining to show insulin (red), EdU (green) and DAPI (blue) in fetal ovine pancreatic islets cultured in 10 ng ml^−1^ IGF‐I (positive control) or 0, 0.1, 1.0 and 10 ng ml^−1^ leptin. Scale bar = 25 μm. *B*, mean (±SEM) proliferation rates in beta cells after incubation with increasing doses of leptin compared to 10 ng ml^−1^ IGF‐I. Values with different letters are significantly different from each other, one‐way ANOVA, *P* < 0.05.

In experiments *in vitro*, islets cultured in complete media without the addition of hormones (basal condition) or with 10 ng ml^−1^ IGF‐I (positive control) showed beta cell proliferation rates of 5.7 ± 0.2 and 8.0 ± 0.4%, respectively (Figs 2, 3, 4).

#### T_3_

Increasing concentrations of T_3_ caused a significant reduction in the number of EdU/insulin‐positive cells in isolated fetal ovine islets (*P* < 0.05; Fig. 2). The rate of beta cell proliferation was decreased significantly by 0.1, 1 and 10 ng ml^−1^ T_3_, in a dose‐dependent manner (Fig. 2 *B*).

#### Insulin

Insulin treatment at 0.1 and 1 ng ml^−1^ did not affect the number of EdU/insulin‐positive cells in fetal pancreatic islets (Fig. 3). At the higher dose of 10 ng ml^−1^ insulin, however, beta cell proliferation rate was increased significantly compared to the basal condition and lower doses of insulin (*P* < 0.05; Fig. 3 *B*), and tended to be higher than that induced by 10 ng ml^−1^ IGF‐I (*P = *0.07; Fig. 3 *B*).

#### Leptin

Incubation with leptin induced a bimodal response in beta cell proliferation (Fig. 4). A significant decrease in the number of EdU/insulin‐positive cells was observed after incubation with 0.1 and 1 ng ml^−1^ leptin (*P* < 0.05; Fig. 4 *B*). However, at the highest concentration, 10 ng ml^−1^ leptin stimulated beta cell proliferation significantly above that seen in the basal condition (*P* < 0.05; Fig. 4 *B*) and similar to that stimulated by IGF‐I (Fig. 4 *B*).

## Discussion

This study has shown for the first time, and contrary to the original hypothesis, that thyroid hormone deficiency in the ovine fetus caused a 30–40% increment in pancreatic beta cell mass, secondary to beta cell hyperplasia, and a concomitant rise in circulating insulin concentration *in utero*. In support of these findings *in vivo*, physiological concentrations of T_3_ reduced, while insulin stimulated, beta cell proliferation rates in isolated fetal ovine pancreatic islets studied *in vitro*. Plasma leptin concentration was increased by fetal hypothyroidism and pancreatic beta cell proliferation showed a bimodal response to leptin exposure *in vitro*. Therefore, in the developing pancreas, beta cell mass is sensitive to variations in circulating thyroid hormones, insulin and leptin before birth.

Hypothyroidism is associated with asymmetric intrauterine growth retardation in this and previous studies (Fisher & Polk, 1989; Kilby *et al*. 1998; Lanham *et al*. 2011). Although the thyroid‐deficient ovine fetus had shorter limbs and reduced weights of the heart, lungs and gastrointestinal tract, other tissues such as the kidneys and perirenal adipose tissue were enlarged by hypothyroidism *in utero*. Before birth, insulin promotes growth of the axial skeleton and tissues such as adipose tissue (Fowden *et al*. 1989), so increased circulating concentrations of insulin seen in the hypothyroid sheep fetus may contribute to maintenance of vertebral length and tissue‐specific patterns of organ growth. The extent to which growth patterns during hypothyroidism relate to changes in insulin signalling pathways in these fetal organs, however, remains to be established.

In hypothyroid fetal sheep, beta cell mass was increased at both gestational ages studied, without any change in absolute or relative mass of the pancreas, although beta cells were only ∼3–7% of the total pancreas mass. Thyroid hormone deficiency *in utero* appears to induce a beta cell‐specific phenotype as absolute and relative alpha cell mass was unchanged. The mechanisms by which hypothyroidism influences development of the endocrine pancreas during late gestation are largely unknown, and may involve beta cell neogenesis from pancreatic progenitor cells and/or replication of pre‐existing beta cells (Bouwens *et al*. 1997). In the pancreas of the human and ovine fetus, primitive islets and insulin production first appears at around 25% of gestation, and, thereafter, beta cells proliferate and secrete insulin throughout gestation (Fowden & Hill, 2001). Suppression of apoptosis may also contribute to alterations in pancreatic beta cell mass *in utero*, although apoptotic cells comprise only ∼2% of the total beta cell population in the intact sheep fetus during late gestation (Limesand *et al*. 2005). In humans, there is a wave of apoptosis in pancreatic islets over the perinatal period in association with a decline in beta cell proliferation (Kassem *et al*. 2000). The prepartum surge in circulating thyroid hormones may therefore have a role in this critical period of islet remodelling. Previous studies have shown that thyroid hormone receptors TRα1 and TRβ1 are important for normal islet development in islets taken from postnatal rodents and in a rat pancreatic beta cell line (Furuya *et al*. 2010; Aguayo‐Mazzucato *et al*. 2013). In contrast to the current findings, however, T_3_ has been shown to stimulate proliferation and reduce apoptosis in pancreatic beta cells *in vitro*, although these studies used islets and cell lines derived from postnatal rather than fetal animals (Verga Falzacappa *et al*. 2006, 2010; Kim *et al*. 2014). The difference in thyroid hormone action between fetal and postnatal beta cells may be due to the relative expression of thyroid hormone transporters and receptors, and the activity of post‐receptor pathways. Thyroid hormone receptor isoforms are expressed in a temporal and tissue‐specific manner during development (Mullur *et al*. 2014), although, to date, differences in the mechanisms of thyroid hormone action have not been characterised in the pancreatic beta cell before and after birth.

While fetal pancreatic beta cell proliferation rates and total mass were influenced by thyroid hormone status, the maturity and functionality of individual beta cells, in terms of their capacity for insulin secretion, are unknown. In fetal sheep, the prepartum surge in T_3_ may promote a switch in pancreatic beta cell development from proliferation and growth to maturation, in a manner similar to that seen in cardiomyocytes near term (Chattergoon *et al*. 2012, 2014). Beta cells in the ovine fetal pancreas become more responsive to glucose and amino acids with gestational age, especially over the perinatal period when circulating T_3_ concentrations rise ((Philipps *et al*. 1978, 1979; Fowden & Hill, 2001). Treatment of immature postnatal rat islets with T_3_ *in vitro* increases mRNA and protein levels of musculoaponeurotic fibrosarcoma oncogene family A (MAFA), a transcription factor essential for beta cell maturation, and promotes glucose‐stimulated insulin secretion (Aguayo‐Mazzucato *et al*. 2013). Similar findings are observed in human fetal pancreatic islets and embryonic stem cells differentiated towards beta cells where T_3_ induces the onset of glucose‐sensitive insulin synthesis and secretion (Aguayo‐Mazzucato *et al*. 2015). Therefore, in fetal sheep, removal of the thyroid gland and prevention of the normal rise in T_3_ near term may maintain proliferation of pancreatic beta cells and basal insulin secretion, and yet delay maturation of glucose‐stimulated insulin secretion. Future studies will examine the expression of signalling molecules that regulate beta cell maturation [pancreatic and duodenal homeobox factor‐1 (PDX1), neurogenin 3 (NGN3), MAFA] and sensitivity to glucose (GLUT‐2, glucokinase, ATP‐sensitive potassium and voltage‐dependent calcium ion channels) in fetal ovine pancreatic islets treated with T_3_ *in vitro* and in those extracted from hypothyroid sheep fetuses.

The effects of hypothyroidism on development of the fetal endocrine pancreas may be mediated indirectly by other hormones, such as insulin, leptin and IGFs. The high circulating concentration of insulin seen in the TX fetus may be part of a positive feedback loop, in which hyperinsulinaemia promotes growth of the endocrine pancreas and further insulin secretion. Indeed, beta cell proliferation *in vitro* was stimulated by the highest dose of insulin, which may reflect the local concentration in the islets. In the pancreas of the hypothyroid ovine fetus, therefore, the increase in insulin secretion from the enlarged beta cell mass may drive further beta cell proliferation in a paracrine manner. Indeed, previous studies have shown that insulin activates mammalian target of rapamycin (mTOR) and S6‐kinase via PI‐3‐kinase and mitogen‐activated protein kinase kinase (MEK1) pathways to stimulate pancreatic beta cell proliferation in cell lines derived from fetal mice (Guillen *et al*. 2006). No significant changes in IGF‐I or II were detected in the circulation of the hypothyroid ovine fetus. IGF‐I was a potent stimulus of beta cell proliferation *in vitro*, although local IGF concentrations in the fetal ovine pancreas were not determined and may have been influenced by thyroid hormone deficiency.

The increased relative perirenal adipose mass observed in the hypothyroid ovine fetus was likely to be responsible for the elevated circulating concentration of leptin. In addition, there may be changes in leptin synthesis and secretion from fetal adipocytes, possibly in response to the rise in circulating insulin concentration *in utero*. Indeed, thyroid hormone deficiency in fetal sheep increases leptin mRNA abundance in perirenal adipose tissue (O'Connor *et al*. 2007). Furthermore, adipose leptin mRNA abundance and plasma leptin concentration correlate with plasma insulin levels both in sheep fetuses infused with insulin and in those hyperinsulinaemic following maternal overnutrition (Devaskar & Anthony, 2002; Mühlhäusler *et al*. 2007). Increased plasma concentrations of leptin in the hypothyroid fetus may contribute to the greater pancreatic beta cell mass as the highest dose of leptin promoted beta cell proliferation *in vitro*. Functional long‐form leptin receptors have been identified in pancreatic islets obtained from fetal rats (Islam *et al*. 2000). Previous reports, however, are conflicting about the role of leptin in determining pancreatic beta cell mass, as it has been shown to have both inhibitory and stimulatory effects, depending on the experimental model used (Islam *et al*. 2000; Morioka *et al*. 2007). In fetal rat islets and a rat‐derived beta cell line, leptin stimulates cell division (Islam *et al*. 1997, 2000), and islet size and beta cell number are reduced in neonatal rats treated with a leptin antagonist (Attig *et al*. 2011). In contrast, greater beta cell mass is observed in mice with pancreas‐specific deletion of the leptin receptor, which appears to be due to beta cell hypertrophy rather than to any change in mitotic or apoptotic markers (Morioka *et al*. 2007). The current study is the first to report a dose‐dependent, bimodal response in beta cell proliferation to leptin in fetal ovine pancreatic islets *in vitro*. The relationship between leptin and beta cell proliferation may arise from changes in the expression and sensitivity of the leptin receptor to varying concentrations of leptin. Several intracellular signalling pathways have been shown to mediate the actions of leptin (Frühbeck, 2006), which may be modulated differentially by the dose of leptin *in vitro*. Taken together, the current findings demonstrate that T_3_, insulin and leptin all contribute to the regulation of fetal beta cell mass in normal development and during hypothyroidism.

The effects of hypothyroidism on development of the fetal endocrine pancreas may have short‐ and long‐term consequences for growth and metabolism in the offspring. Adult offspring of rats hypothyroid during pregnancy show reduced glucose tolerance and lower glucose‐stimulated insulin secretion from isolated pancreatic islets (Farahani *et al*. 2010; Karbalaei *et al*. 2014). Although these offspring were euthyroid in adult life, the pancreatic islets were less sensitive to glucose with lower insulin output per islet (Karbalaei *et al*. 2014). The beta cell mass of these offspring was not determined at birth or in adult life, and there may be dynamic changes in islet morphology and function over the perinatal period and into adulthood.

In summary, this study shows that thyroid hormones play a central role in controlling pancreatic beta cell mass in the ovine fetus during late gestation. From studies both *in vivo* and *in vitro*, the effects of hypothyroidism on pancreatic beta cell proliferation and mass before birth appear to be due not only to the deficit in circulating thyroid hormones, but also to coincident changes in insulin and leptin exposure. Whether prepartum changes in thyroid hormones influence the functional maturity of beta cells at birth with consequences for glucose‐sensitive insulin secretion and the development of diabetes in later life remains to be determined.

## Additional information

### Competing interests

None.

### Author contributions

All studies *in vivo* were carried out at the University of Cambridge followed by experimental work and analysis at Oxford Brookes University; the studies on beta cell proliferation *in vitro* were carried out at the University of Arizona. Conception and design of the experiments: S.E.H., D.M., A.L.F., S.W.L. and A.J.F. Collection, assembly, analysis and interpretation of data: S.E.H., M.J.D., M.A.D., A.K., H.M.D., F.B.P.W., D.B., D.M., M.A., A.L.F., S.W.L. and A.J.F. Drafting the article: S.E.H. and A.J.F. All authors approved the final draft of the manuscript and all persons designated as authors qualify for authorship.

### Funding

The project was funded in part by the Biotechnology and Biological Sciences Research Council (BB/HO1697X/1). S.E.H. was supported by a Nigel Groome PhD Studentship at Oxford Brookes University. S.E.H. was awarded a practical skills grant from the Society for Endocrinology and a travel grant from the Physiological Society to fund work at the University of Arizona.

**Translational perspective**

Development of pancreatic beta cell mass and insulin secretion is essential for normal fetal growth and carbohydrate metabolism before and after birth. Thyroid hormones are also important regulators of growth and development in the fetus. Therefore, using the fetal sheep model, it was hypothesised that growth retardation associated with thyroid hormone deficiency is due to reductions in pancreatic beta cell mass and circulating insulin concentration *in utero*. Contrary to the original hypothesis, greater beta cell proliferation and mass, and elevated plasma insulin concentration, were observed in hypothyroid sheep fetuses. Hypothyroidism *in utero* also caused an increase in perirenal adipose tissue mass and plasma leptin concentration. In isolated ovine fetal pancreatic islets, beta cell proliferation was decreased by thyroid hormone in a dose‐dependent manner and stimulated by high concentrations of insulin and leptin. Overall, these studies *in vivo* and *in vitro* show novel endocrine interactions in the control of pancreatic beta cell mass before birth. Future studies will explore the functional capacity of fetal beta cells exposed to varying concentrations of thyroid hormones, insulin and leptin, and will characterise the molecular mechanisms underlying the endocrine control of beta cell proliferation and maturation *in utero*. The finding that thyroid hormones influence beta cell development in the fetus has important implications for growth and carbohydrate metabolism in infants affected by congenital hypothyroidism and maternal thyroid hormone disorders during pregnancy. In individuals hypothyroid before birth, abnormalities in beta cell mass may predispose to pancreatic dysfunction and type 2 diabetes in later life.
